# Dietary Interventions in Multiple Sclerosis: Development and Pilot-Testing of an Evidence Based Patient Education Program

**DOI:** 10.1371/journal.pone.0165246

**Published:** 2016-10-20

**Authors:** Karin Riemann-Lorenz, Marlene Eilers, Gloria von Geldern, Karl-Heinz Schulz, Sascha Köpke, Christoph Heesen

**Affiliations:** 1 Department of Neurology, Institute for Neuroimmunology and Multiple Sclerosis, University Medical Center Hamburg-Eppendorf, Hamburg, Germany; 2 Department of Neurology, University of Washington, Seattle, Washington, United States of America; 3 Department of Psychosocial Medicine, Institute of Medical Psychology and Athleticum—Competence Center for Sports- and Exercise Medicine, University Medical Center Hamburg-Eppendorf, Hamburg, Germany; 4 Institute of Social Medicine and Epidemiology, Nursing Research Unit, University of Lübeck, Lübeck, Germany; 5 Department of Neurology, MS Day Hospital and Outpatient Unit and Institute for Neuroimmunology and Multiple Sclerosis, University Medical Center Hamburg-Eppendorf, Hamburg, Germany; Waseda University, JAPAN

## Abstract

**Background:**

Dietary factors have been discussed to influence risk or disease course of multiple sclerosis (MS). Specific diets are widely used among patients with MS.

**Objective:**

To design and pilot-test an evidence based patient education program on dietary factors in MS.

**Methods:**

We performed a systematic literature search on the effectiveness of dietary interventions in MS. A web-based survey among 337 patients with MS and 136 healthy controls assessed knowledge, dietary habits and information needs. An interactive group education program was developed and pilot-tested.

**Results:**

Fifteen randomised-controlled trials (RCTs) were included in the systematic review. Quality of evidence was low and no clear benefit could be seen. Patients with MS significantly more often adhered to a `Mediterranean Diet`(29.7% versus 14.0%, p<0.001) compared to controls. 143 (42%) of the patients with MS had tried special MS diets. Important information needs addressed effectiveness of MS diets (44%) and relation between nutrition and MS (43%). A pilot test of our newly developed patient education program with 13 participants showed excellent comprehensibility and the MS-specific content was judged as very important. However, the poor evidence base for dietary approaches in MS was perceived disappointing.

**Conclusions:**

Development and pilot-testing of an evidence-based patient education program on nutrition and MS is feasible. Patient satisfaction with the program suffers from the lack of evidence. Further research should focus on generating evidence for the potential influence of lifestyle habits (diet, physical activity) on MS disease course thus meeting the needs of patients with MS.

## Introduction

Multiple sclerosis (MS) is a demyelinating disease and the most common cause of neurological disability in young adults worldwide. [1, 2] The etiology of multiple sclerosis–especially the pathophysiological mechanisms of neurodegeneration which cause irreversible disability—are still largely unknown. [1, 3] Although by now more than a hundred genes are known to increase the risk of MS, all of them only contribute marginally. [4] This as well as the results of twin studies support the premise that, apart from genetic factors, environmental influences modify disease risk and progression, possibly through epigenetic changes which could up- or downregulate immune response and influence neural development. [1, 5] Environmental factors that have been proposed to influence disease risk or progression are infection with Epstein-Barr virus, smoking, low Vitamin D status, and a variety of dietary factors such as high sodium and unsaturated fatty acid intake. [1, 5, 6] Recently, increased attention among researchers has been paid to salt restriction [7], ketogenic diet [8] and the relevance of gut microbiota. [9]

However, high level evidence from randomized controlled trials on the influence of dietary factors on disease progression is scarce. Jagannath et al. (2010) [10] only reported on one small trial with 49 participants in their Cochrane Review on Vitamin D treatment in MS. Another Cochrane Review from 2012 included all randomised or controlled clinical trials on specific dietary interventions, diet plans or dietary supplementation except for vitamin D supplementation. [6] They concluded that polyunsaturated fatty acids do not seem to have major clinical effects on disease progression, but may be associated with a tendency in reduction of frequency of relapses over two years. However, data available were insufficient to assess real benefit or harm, because of poor trial quality. [6]

Although evidence on the efficacy of dietary interventions is poor, patients with MS (PwMS) have a strong interest in dietary recommendations and many adhere to special diets. There is a large number of books and webpages which support multiple, sometimes conflicting approaches. [11] An internet search using the terms ‘diet’ and ‘multiple sclerosis´ carried out by Farinotti et al. in 2012 produced over 27 million links. [6] A German survey among 1573 PwMS showed that the lifetime use of dietary modification was 41%. [12] An Australian survey with 416 PwMS found that a high percentage of participants took supplements (63.2%) and/or followed special dietary recommendations. [13]

As the nutritional knowledge of PwMS as well as their interest in understanding diet and nutrition are largely unknown, we aimed to better understand these factors. Being aware of the large gap between scientific evidence on the effectiveness of dietary interventions in MS and dietary behaviour of PwMS, we hypothesized that there is a need for an evidence based patient education program. Accordingly our aim was to design and pilot-test an evidence based patient education program on the influence of diet on MS.

## Methods

### Systematic literature search

We performed a systematic literature search in PubMed in September 2010 with an update in June 2015 on the topics MS and dietary interventions (full search terms see S1 Text). We defined inclusion criteria listed in Table 1.

**Table 1:** Eligibility criteria used for literature search and screening.

Population	PwMS (all types)
Intervention	Any dietary intervention
Control	Placebo/ other control intervention
Outcome	Patient relevant outcome: health related quality of life, disease activity, relapse rate, disability, fatigue
Study types	RCT, CCT; Study duration ≥ 1 year; Total number of participants ≥ 30
Publication Language	English or German
Publication	Full-text publication available/ procurable

PwMS = patients with MS, RCT = Randomized controlled trial, CCT = Controlled clinical trial.

We assessed the risk of bias of the included studies using the Cochrane risk of bias assessment tool.[14]

### Web-based survey

We developed a questionnaire and performed a web-based survey among PwMS and healthy controls. Visitors of the website of the German Multiple Sclerosis Society (DMSG) were invited to the survey in spring 2011. The link on the DMSG website directed participants to the web-based questionnaire. Informed consent was obtained via the first page of the questionnaire. In order to recruit participants for a control group, an additional call for participation was put on the DMSG website addressing friends and relatives of PwMS.

#### Survey questionnaire

The questionnaire consisted of four parts with a total of 36 items, 31 of which were newly developed by the research team. Most of the questions were directed to PwMS and control group participants but some could only be answered by PwMS. The questionnaire covered the following topics.

Part 1 (12 items): Knowledge and Attitudes towards Nutrition/Diet. Questions addressed beliefs about the influence of different lifestyle factors (e.g. stress, sports) on MS. Patients could attribute 0 to 100 points to the different factors, 0 meaning no influence, 100 meaning maximum influence. Additionally we assessed knowledge about MS diets, motives for adherence to a diet, self-perceived effects of dietary behaviours and we asked about the use of different sources of information.

Part 2 (17 items): Dietary Habits. Questions addressed intake of supplements and adherence to MS-specific diets. Eating habits in general were assessed based on a questionnaire from the `Genes and environment in MS (GEMS)`project. [15] Participants were asked to select a diet that best fit their usual dietary habits (see Table 2).

**Table 2:** Categories of types of diets pre-specified in the survey.

**Mixed Diet**: All sorts of meat or fish, vegetables, fruits, milk products and grain
**Mediterranean Diet**: Diet with great amounts of fruits, vegetables, lean meat, fish, seafood, olive oil and nuts. Reduced intake of butter and cream.
**Vegetarian diet with fish and seafood**: Vegetarian diet including eggs, milk products as well as fish and seafood.
**Vegetarian diet** including eggs and milk products
**Vegan diet**: Vegetarian diet without any animal protein
**Glycemic Index Diet**: Diet with fixed glycemic index, including little or no short chain carbohydrates
**Other diets** (free text answers)

As the absolute number of responses was low, we combined the three types of vegetarian diets into the category `Vegetarian Diet´ for data analysis. `Glycemic Index Diet´ and Other Diets´ were put together into the category `Other Forms of Diet´.

Part 3 (5 items): Physical Activity Assessment with the “Godin Leisure Time Exercise Questionnaire”[16, 17] and one additional item. Data not shown.

Part 4 (2 items): Patients´ Interests and Preferences regarding different topics for a patient education program on diet and multiple sclerosis. We presented six possible major themes and participants were asked to rank them from 1 = most important to 6 = less important. An open question gave patients the possibility to name additional topics of interest.

Additionally, we asked for socio-demographic data, type and course of disease, weight and smoking status. Mental health status was assessed with 2 questions about depression from Mohr et al., 2007 [18] and self-efficacy with 5 questions from Schwarzer et al., 1999 [19] (data not shown).

### Development, pilot-testing and evaluation of the education program

Based on the results of the systematic literature search and the results of the survey, a group-education program was developed in accordance with the concept of evidence-based patient information [20, 21] (see also S2 Text) and the Medical Research Council (MRC) framework on the development and evaluation of complex interventions. [22] Patients from the MS Day Hospital were invited to participate in pilot training sessions. The first session was conducted in May 2011 with 4 participants. After revision of the program according to the feedback of the first group, the second training session was conducted with 9 participants.

Directly after the education program participants were asked about the extent and quality of the information presented. Using visual analogue scales with a scale from 0 to 10 the following domains were assessed: novelty of information (0 = new, 10 = already known), comprehensibility (0 = comprehensible, 10 = incomprehensible), importance (0 = important, 10 = not important), extent of information (0 = too extensive, 10 = not sufficient) and impact of the information (0 = disappointing, 10 = encouraging). Two open questions asked for the personally most relevant information received and changes that should be made to the training. Additionally, participants were encouraged to express their opinion in an open discussion at the end of the training program.

### Statistical analysis

This survey was conducted using the online software Enterprise Feedback Suite (EFS) Survey 8.0 (Globalpark) and the online platform www.unipark.info. Data were analysed using SPSS 22.0 for Windows. Based on own previous surveys and work of others on lifestyle factors in MS a sample size of at least 100 participants in PwMS and control group was considered sufficient to detect major differences. For dichotomous data we calculated absolute numbers and percentages and performed Pearson’s chi-squared test to test for significance. For continuous data we calculated mean and standard deviation and used the t-test for independent samples to test for significance. Pearson’s chi-squared test was calculated to assess the difference in usual dietary habits and supplement intake between PwMS and the control group.

### Ethical considerations

Ethical approval was provided by the Ethics Committee of the University of Lübeck, Germany (document number 15–264).

## Results

### Results of the systematic literature search

#### Screening process

**Fig 1:**
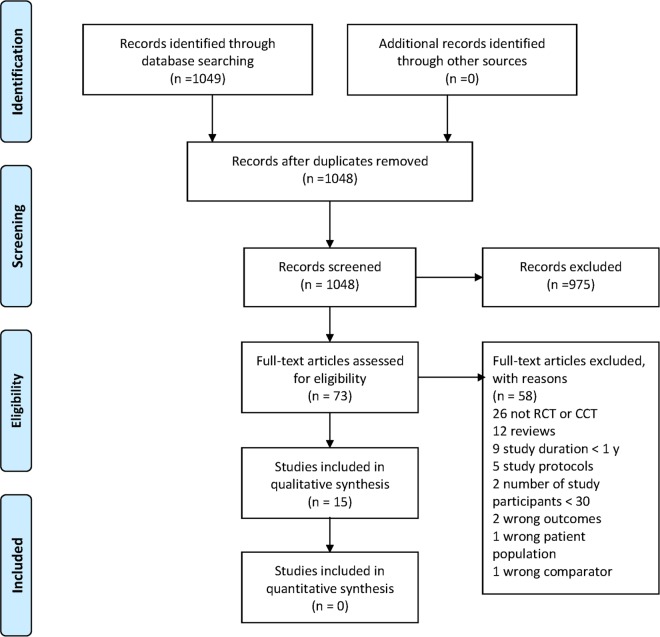
Flow chart of screening process according to PRISMA. [23]

Our search in June 2015 yielded 1048 hits after removal of duplicates. One investigator (KRL) reviewed citations on the basis of title and abstract information and excluded those clearly not meeting inclusion criteria. We retrieved the full text of the remaining studies which were subsequently reviewed independently by two investigators (KRL, SK) to assess whether inclusion criteria were met and to rate quality. All differences were settled by discussion. A flow chart of the screening process is shown in Fig 1. For additional information see S1 Checklist.

We included 9 trials modifying fatty acid intake and 6 trials on Vitamin D. The main characteristics and results of the trials are displayed in Tables 3 and 4.

**Table 3:** Characteristics of included studies–trials modifying fatty acid intake by supplementation.

Study	Year and Country	Design	Intervention	Outcome parameters		Main results (as described by study authors)
Bates, 1977 [24]	ns, UK	Double blind RCT, 24 months, 152 Patients with secondary progressive MS(18 dropouts)	**Treatment A**: 8 capsules of 0.6 ml oil, containing 360 mg linolenic acid and 3.42 g linoleic acid daily **Control B:** 8 capsules of 0.6 ml of oleic acid **Treatment C**: 11.5 g of linoleic acid/d as a spread**Control D**: 4 g of oleic acid/d as a spread	Disability (DSS); relapses (frequency, site, duration, severity)		No significant effect on disability or relapse rate/severity of relapses
Bates, 1978 [25]	ns, UK	Double blind RCT, 24 months, 116 Patients with RRMS (12 dropouts)	**Treatment A**: 8 capsules of 0.6 ml oil, containing 340 mg linolenic acid and 2.92 g linoleic acid daily, **Control B**: 8 similar capsules providing 4 g of oleic acid/d; **Treatment C**: 23 g of linoleic acid/d as a spread; **Control D**: 16 g of oleic acid/d as a spread	Disability (DSS); relapses (frequency, site, duration, severity)		No significant effect on disability status and relapse rate; severity and duration of relapses in favour of intervention C only
Bates, 1989 [26]	ns, UK	Double blind RCT, 24 months, 312 Patients with RRMS (20 dropouts)	**Treatment**: 20 Capsules of 0.5 g `MaxEPA`oil, providing 1.71 g EPA and 1.14 g DHA daily **Control:** 20 Capsules of 0.5 g of olive oil, containing 72% oleic acid. Dietary Advice for both groups to enhance intake of n-6-polyunsaturated fatty acids.		Change in overall disability (DSS); number, duration and severity of relapses	No significant effect on disability and relapses, but a trend towards benefit of omega-3-fatty acids intervention on all parameters
Harbige, 2007 [27]	ns, UK	Double-blind, placebo-controlled RCT, 18 months, 36 PwMS, (8 dropouts)	**Treatment A** (High Dose): 14 g/d GLA-rich borage oil **Treatment B** (Low Dose): 5 g/d GLA-rich borage oil **Control:** Placebo- polyethylene glycol 400		Change in relapse rate (ARR); disability (EDSS)	Significant beneficial effect on ARR and EDSS when comparing high dose to placebo intervention
Millar, 1973 [28]	ns, UK	Double blind RCT, 24 months, 87 PwMS (12 dropouts)	**Treatment:** Sunflower seed oil emulsion: 2 doses of 30 ml providing 8.6 mg linoleic acid each **Control:** 2 doses of 30 ml olive oil providing 3.8 mg oleic acid and 0.2 mg linoleic acid each		Disability (DSS and other measures); relapses	No significant effect on disability status and relapse rate; severity and duration of relapses in favour of intervention
Pantzaris, 2013 [29]	2007–2009, Cyprus	Double blind RCT, 30 months, 80 patients with RRMS (39 dropouts)	**Treatment A:** EPA (1650 mg)/DHA (4650 mg)/ GLA (2000 mg)/LA (3850 mg)/total other Ω-3 (600 mg)†/total MUFA (1714 mg)+total SFA (18:0 160 mg, 16:0 650 mg)/vitamin A (0.6 mg)/ vitamin E (22 mg) plus citrus Aroma **Treatment B:** as A + pure γ-tocopherol (760 mg) plus citrus aroma **Treatment C**: Pure natural γ-tocopherol (760 mg) dispersed in pure virgin olive oil (16137 mg) as delivery vehicle plus citrus aroma **Treatment D (Placebo)**: Olive oil (pure virgin) plus citrus aroma		ARR at 2 years; Time to confirmed disability progression at 2 years (EDSS)	No significant difference in the ARR after 24 months. Cumulative probability of progression was 10% in Treatment Group B and 35% in the placebo group (p = 0.052).
Paty, 1983 [30]	ns, Canada	Double-blind RCT, 30 months, 96 PwMS (20 dropouts)	**Treatment:** Sunflower seed oil emulsion (66.2% linoleic acid) yielding a dosage of 17 g/day of linoleic acid **Control**: Olive oil emulsion (83.5% oleic acid, 4% linoleic acid) yielding a dosage of 21 g/day of oleic acid		Disability (DSS); timed functional studies.	No benefit from the use of linoleic acid (no information about statistical significance given)
Torkildsen, 2012 [31]	2004–2008, Norway	Double blind RCT, 24 months, 92 patients with RRMS (11 dropouts)	**Treatment:** 5 capsules of 1 g `Triomar`providing a total of 1350 mg EPA and 850 mg DHA/d. 4 IU of α-Tocopherol per gram for anti-oxidative protection. **Control:** 5 capsules with corn oil/d. After 6 months all patients received 44 μg of interferon beta-1a 3 times per week subcutaneously for another 18 months.		MRI disease activity, relapse rate, disability, fatigue, quality of life, safety	No significant difference in relapse rate, disability progression (EDSS), Multiple Sclerosis Functional Composite scores, Fatigue Severity Score or SF-36 after 6 and 24 months
Weinstock-Guttman (2005) [32]	ns, USA	Double blind RCT, 12 months, 31 PwMS on DMT (10 dropouts)	**Treatment**: low fat diet (< 15% fat) with 6 capsules of Omega-3- fatty acids providing 1.98 g EPA and 1.32 g DHA daily. (FO-Group) **Control:** AHA Step 1 diet (total fat ≤30%; saturated fat < 10%) and 6 capsules of 1 g Olive Oil daily (OO-Group). All patients: 400 IU Vitamin E/d, 500 mg Calcium/d and a multivitamin tablet. Dietary advice to meet the fat intake recommendations.		Primary: Physical Component Scale (PCS) of the SF-36; Secondary: Modified Fatigue Impact Scale (MFIS) and Mental Health Inventory (MHI); relapse rate; disability (EDSS)	Significant benefit in PCS/SF-36 and MHI scale for the Treatment Group at 6 months, but not at 12 months. Significant benefit in MFIS at 6 months for the Control Group and trend maintained at 12 months. Reduced relapse rate for both groups compared to the year prior to the study. Weak trend towards an increase in EDSS in the OO group versus a decrease FO group.

AHA = American Heart Association; ARR = Annualized Relapse Rate; DHA = Docosahexaenoic Acid; DMT = Disease Modifying Treatment; DSS = Disability Status Scale; EDSS = Expanded Disability Status Scale, EPA = Eicosapentaenoic Acid, GLA = Gamma Linolenic Acid; IU = International Units; ns = not specified; MRI = Magnetic Resonance Imaging; MUFA = Mono unsaturated fatty Acids; PwMS = Patients with MS; RCT = Randomized Controlled Trial, RRMS = Relapsing Remitting Multiple Sclerosis, SFA = Saturated fatty Acids.

**Table 4:** Characteristics of included studies–trials modifying Vitamin D intake by supplementation.

Study	Year and Country	Design	Intervention	Outcome parameters	Main results (as described by study authors)
Burton, 2010 [33]	2006–2008, Canada	Open-label RCT, 12 months, 49 PwMS (4 dropouts)	**Treatment:** Escalating doses of Vitamin D3 up to 40000 IU/d plus 1200 mg Ca/d. **Control** patients were permitted to take 4000 IU/day of vitamin D and supplemental calcium if desired.	Safety/adverse events; relapse rate (ARR), disability progression (EDSS)	No biochemical or clinical adverse events reported; Trend for treatment benefit in ARR and EDSS score, but not statistically significant compared to control group
Derakshandie, 2013 [34]	2010–2011, Iran	Double blind RCT, 12 months, 30 patients with Optic Neuritis (ON), no MS, 25-OH-Serum level < 30 ng/ml, (6 dropouts)	**Treatment**: Oral Vitamin D3 at a dosage of 50000 IU/week. **Control:** Placebo	Optic neuritis conversion rate to MS; T1 and T2 brain MRI lesions	**Per Protocol Analysis only:** 5 of 11 (45.5%) control patients and 0 of 13 patients in the treatment group progressed to MS.
Golan, 2013 [35]	2010–2011, Israel	Double blind RCT, 12 months, 45 patients with RRMS on IFN-ß-Therapy and with 25-OH-serum level < 75 nmol/l (15 dropouts)	**Treatment A:** Low dose group: 800 IU Vitamin D3/d. **Treatment B**: High dose group: 4370 IU Vitamin D3/d.	Flu-like symptoms, relapses, disability progression (EDSS), quality of life, adverse events	No significant effect on relapse rate, EDSS, quality of life, or Flu-like symptoms. No major adverse events observed.
Kampman, 2012 [36]	2007–2010, Norway	Double-blind, placebo-controlled RCT, 96 weeks, 71 patients with RRMS, (4 dropouts)	**Treatment:** 20000 IU Vitamin D3 (Cholecalciferol) once a week as a capsule and 500 mg Calcium/d. **Control**: Placebo capsule and 500 mg Calcium/d	Primary: bone mineral density in PwMS; Secondary: relapses (ARR), disability (EDSS), MSFC, grip strength and fatigue.	No significant effect on relapse rate, disability progression, functional tests or fatigue severity.
Shaygannejad, 2012 [37]	2007–2009, Iran	Double-blind, placebo-controlled RCT, 12 months, 50 patients with RRMS on DMT, (0 dropouts)	**Treatment**: 0.5 μg Calcitriol/d administered as capsules twice a day. **Control**: Placebo capsules	Number of relapses, relapse rate, disability progression (EDSS), adverse events	No significant difference in relapse rate or EDSS score between treatment and placebo group. No unexpected safety risks.
Soilu-Hänninen, 2012 [38]	ns, Finland	Double-blind, placebo-controlled RCT, 12 months, 66 patients with RRMS on interferon ß-1b treatment, (2 dropouts)	**Treatment:** 20000 IU Vitamin D3 once a week per oral as add on treatment to interferon ß-1b. **Control**: Placebo capsule	Primary: T2 burden of disease on MRI scans, number of adverse events; Secondary: Number of MRI enhancing T1 lesions and new T2 lesions, relapse rate (ARR), disability progression (EDSS), timed 25 foot walk test and timed 10 foot tandem walk test	Statistically significant greater reduction in number of T1 enhancing lesions in treatment group. No significant differences in EDSS, ARR, walk test results and adverse events

ARR = Annualized Relapse Rate; DMT = Disease Modifying Treatment; DSS = Disability Status Scale; EDSS = Expanded Disability Status Scale, IU = International Units; ns = not specified; MRI = Magnetic Resonance Imaging; MSFC = Multiple Sclerosis Functional Composite; PwMS = Patients with MS; RCT = Randomized Controlled Trial, RRMS = Relapsing Remitting Multiple Sclerosis.

#### Risk of bias of included studies

**Fig 2:**
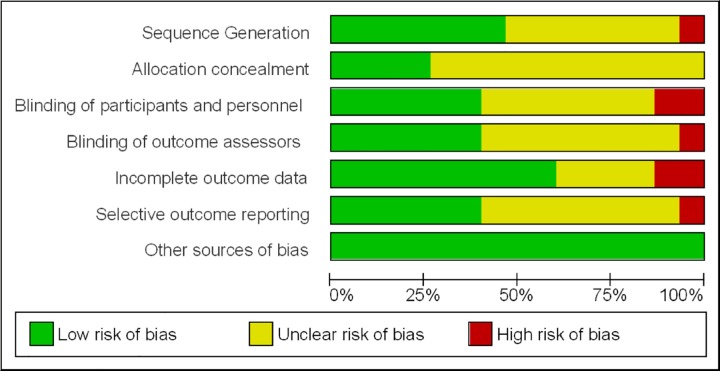
Overall risk of bias across all included studies as judged by the reviewers. Note: `Other sources of bias`are relevant in certain circumstances, relating mainly to particular trial designs (e.g. carry-over in cross-over trials and recruitment bias in cluster-randomized trials). [14].

**Fig 3:**
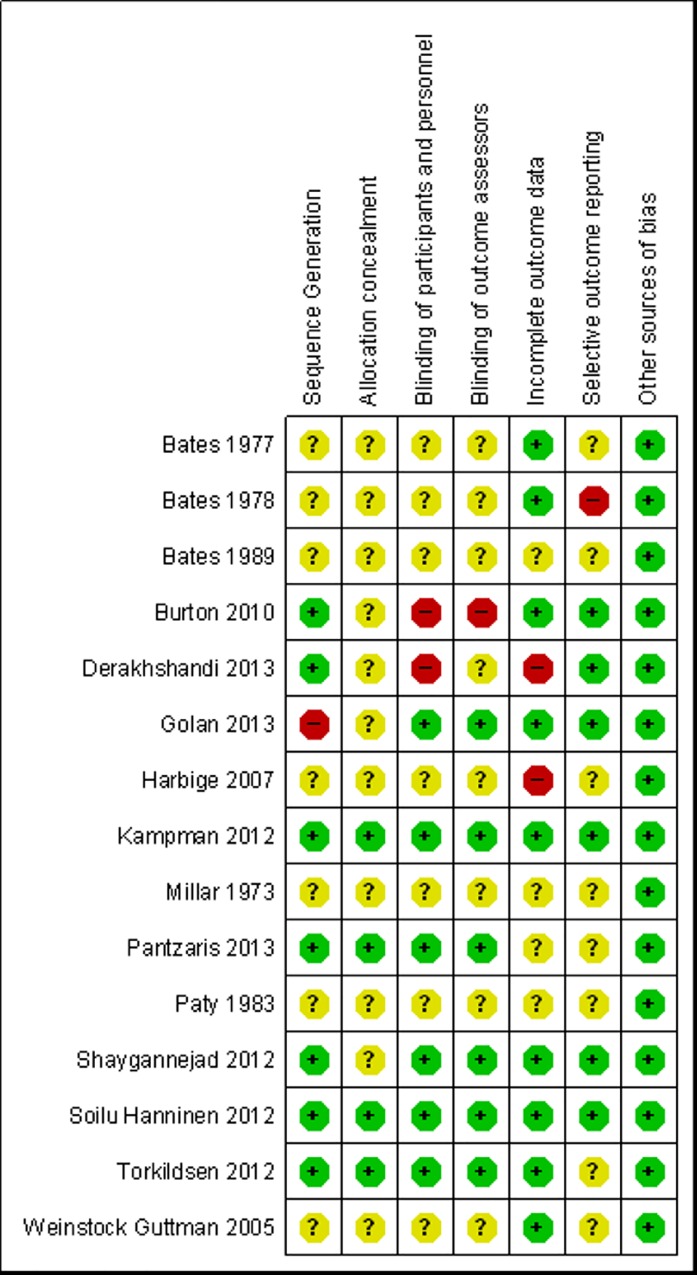
Risk of bias of included studies as judged by the reviewers. Note: `Other sources of bias`are relevant only in certain circumstances, relating mainly to particular trial designs (e.g. carry-over in cross-over trials and recruitment bias in cluster-randomized trials). [14].

The risk of bias of included studies generally was substantial (Fig 2) with only two recent Vitamin D trials being judged as of low of risk of bias (Fig 3).

Most trials could not show significant effects of supplementation considering patient relevant outcomes like disease progression or relapse rate (see Tables 3 and 4). This is in line with the findings from previous systematic reviews. [6, 10]

### Results of the web-based survey

#### Socio-demographics

Between May and July 2011, 583 persons read the information sheet for the survey and 550 (94.3%) actually started the survey. 473 (86%) participants completed the survey, 337 PwMS and 136 healthy controls. Socio-demographic characteristics were comparable between patients and controls except for the age, which was significantly lower in the control group (Table 5).

**Table 5:** Sociodemographic data of PwMS and controls.

	PwMS	Controls	p-value
Number	337	136	
Gender Female, number (%)	238 (71)	97 (71)	0.88
Age (mean±SD)	39 ±10.5	35 ±12.5	0.006
BMI (kg/m^2^, (mean±SD)	24.2 ± 4.5	23.6 ± 3.9	0.14
Smokers, number (%)	80 (23.7)	28 (20.6)	0.46
Years since first symptoms (mean±SD)	10.3 ± 8.4	n.a.	-
Years since diagnosis (mean±SD)	6.8 ± 6.6	n.a.	-

n.a. = not applicable.

#### Dietary habits

**Fig 4:**
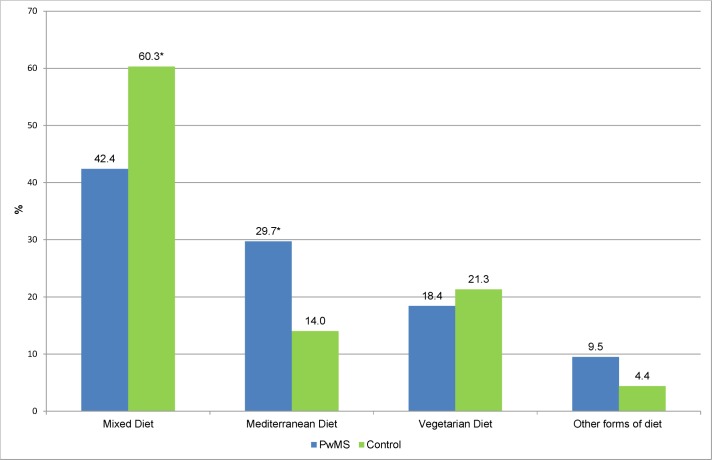
Type of diet among PwMS and controls in % (n = 473). * = significant difference (p<0.001) PwMS = Patients with MS.

PwMS more often adhered to a `Mediterranean Diet`(29.7% versus 14.0%, p<0.001). In contrast controls more often stated, that they followed a `Mixed Diet´ (60.3% versus 42.4%, p<0.001) as shown in Fig 4.

#### Adherence to MS diets or special dietary recommendations

**Fig 5:**
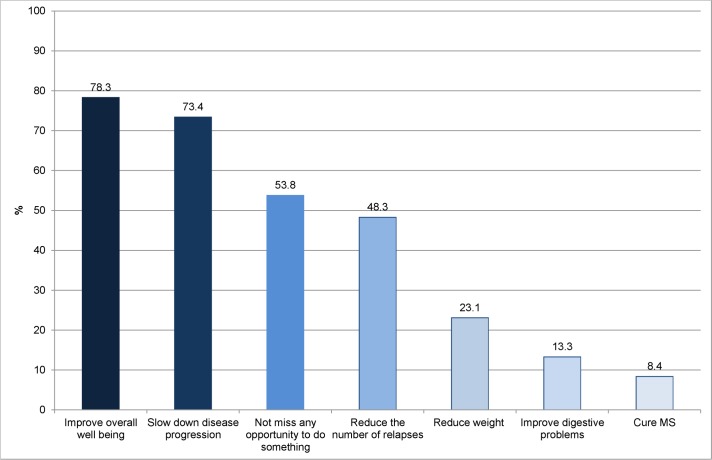
Patients’ goals of using specific diets. (n = 143) (Multiple answers possible).

42% of the patients in our survey indicated that they had already tried to follow an MS diet or had adhered to special dietary recommendations for PwMS. Of these 143 PwMS who followed dietary recommendations, 36 (25%) tried to modify fatty acid intake (less saturated fat, more unsaturated fatty acids), 30 (21%) ate no meat, 31 (22%) adhered to a vegetarian diet (some of them including fish) and 24 (17%) indicated that they tried to eat less or no meat and more fish. Several other diets that were occasionally mentioned included diets according to the principles of Swank [39], Jelinek [40], Adam [41], Evers [42], Kluge [43], a low-carb-diet, paleo diet, and diets without sugar or food additives. Patients who adhered to dietary recommendations (n = 143) reported on their goals from a list of possible objectives (Fig 5).

#### Intake of dietary supplements

Dietary supplements were very commonly used in our sample. About three quarters (76%) of PwMS as well as control group participants indicated, that they had used dietary supplements. In both groups a wide range of vitamins (multivitamins, C, D, E, folate, other vitamins of the Vitamin-B-group), minerals (calcium, magnesium, copper, iron) and other nutritional supplements (e.g. gingko, green tee extract, Indian psyllium seed) were used. However, there were relevant differences in the frequency of use of different supplements between PwMS and controls. PwMS used Vitamin D (PwMS 62%, controls 40%) and omega-3-fatty acids (PwMS 67%, controls 31%) significantly more often than controls, whereas controls used Vitamin C (PwMS 60%, controls 82%) significantly more often (p<0.001). Selenium was used more often by PwMS and controls tended to use zinc more often, but these differences were not statistically significant.

As adhering to a diet might be a challenge, we asked PwMS how they felt following dietary recommendations and if they had ever interrupted their diet. Interestingly, only about 25% of PwMS reported that they had stopped their diet and about 67% reported that they felt better (49%) or even clearly better (18%) when being on a diet. 30.1% of PwMS felt unchanged. Those who stopped their diet (n = 36) mainly reported the following reasons: too restrictive, no effect, too much effort, too expensive (for details see S1 Table).

#### Perceived influence of diet and other lifestyle factors on MS

Diet is clearly a factor many patients in our survey perceived to impact the development and disease course of MS. About one third believed that dietary factors influence the development of multiple sclerosis and about 60% believed that the course of disease can be influenced by dietary factors.

Asked retrospectively, the importance diet/nutrition had for PwMS increased after they were diagnosed with MS. Before diagnosis about 54% of respondents considered diet/nutrition as important (13.1%) or rather important (40.7%), whereas after diagnosis this number increased to about 82% (important: 46.3%, rather important: 35.3%).

**Fig 6:**
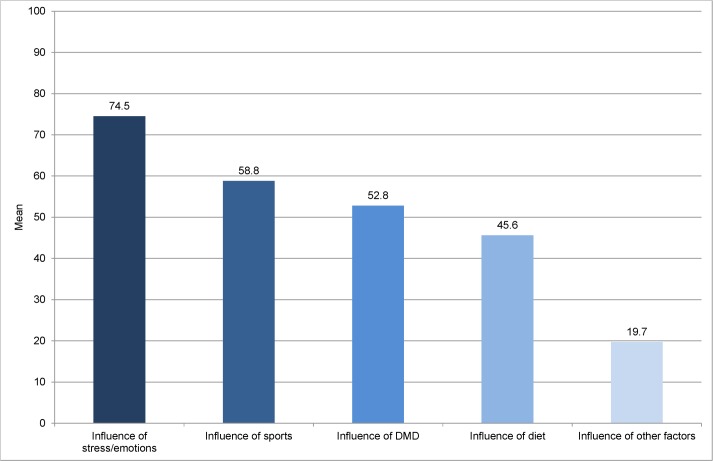
Influence of different factors on disease course in the perception of PwMS. Patients could attribute 0 to 100 points to the different factors, 0 meaning no influence, 100 meaning maximum influence. n = 337 DMD = Disease Modifying Drugs.

However, diet is only one and not the most important influencing factor on disease course in the perception of PwMS. Respondents perceived the influence of stress/emotions, physical activity and Disease Modifying Drugs (DMD) to be larger than the influence of diet (Fig 6).

#### Information seeking behaviour

Only 9.5% of PwMS in our sample never sought information about dietary recommendations for MS. In contrast 43% thoroughly looked for such advice and 47.5% sought some information. Major source of information was the internet (83%), followed by books (64.3%) and physicians (36.7%). Other patients (23.6%), hospitals/rehabilitation centres (23%) and self-help organisations (12.1%) played a minor role in getting information on dietary advice. When asked about their knowledge about special MS diets, most patients had heard about the Evers diet (48.4%) [42], followed by the Fratzer diet (28.5%) [44], gluten-free diet (26.1%) and the Swank diet (20.8%). [39] About one third of PwMS (37.1%) had never heard of any of the special MS diets mentioned in the survey.

#### What information on diet and MS do patients perceive as most relevant

**Fig 7:**
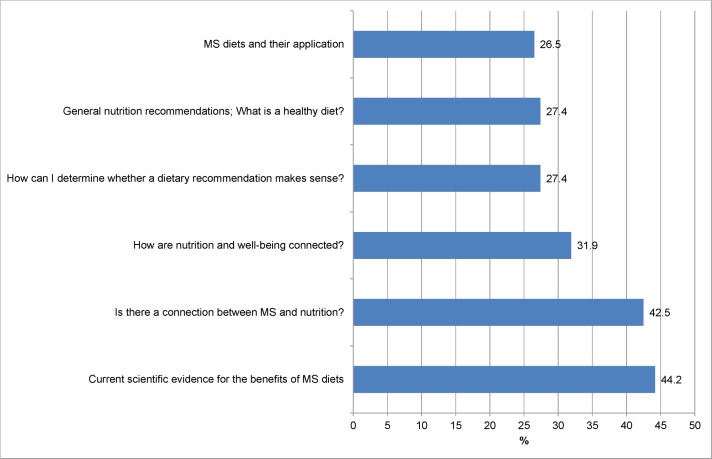
Importance of different topics in a patient education program. Percentage of PwMS assigning rank 1 or 2 to the topic (n = 113).

Participants were asked which topics would be of greatest interest to them for an education program on diet and MS. Unfortunately, the request to assign a hierarchy of importance to the presented themes was not understood by a large proportion of respondents (66.5%) leading to wrong answering patterns. Therefore results of this question can only be displayed for 113 PwMS as shown in Fig 7.

The most important topic to PwMS in our survey was to get information about”Current scientific evidence for the benefits of MS diets“(44.2% of PwMS assigned rank 1 or 2 to this topic) closely followed by the question if “there is a connection between MS and nutrition” (42.5%). However, the other topics were also of interest for at least a quarter of PwMS in our survey.

### Patient education program–pilot study

#### Structure and content

The education program lasted 2 hours and was conducted by a medical student with an interest in nutrition sciences. It consisted of power point presentations on the content shown in Table 6. Within the program there were two short group discussions, one to work on understanding the quality of studies and one opportunity for sharing their experiences with MS diets, as well as a final group discussion.

**Table 6:** Content of the education program.

**Part 1**
Introduction to the aims and structure of the education program
Epidemiology of MS and associations with non-genetic risk factors including diet
Basic knowledge on different study designs (observational and intervention studies), their inherent methodological problems and quality criteria
Endpoints of MS studies (e.g. EDSS, relapse rate, surrogate measures) and associated problems
Group exercises on study quality (e.g. controlled versus uncontrolled studies, sample size etc.)
**Part 2**
Sharing of experiences with MS diets
Introduction to common MS diets (Evers, Fratzer, Swank [42,44,39]
Randomized controlled trials studying diet and MS and their results
Final discussion

#### Pilot study results

The cohort consisted of 13 participants (including two accompanying persons) in two sessions (four and nine participants respectively) with a mean age of 38.5 years (±SD 12.3). Ten participants were female, three were male. The results of the program evaluation for PwMS are displayed in Table 7.

**Table 7:** Pilot evaluation of the program by 11 PwMS; Median and Range (0–10) are displayed.

	Median (Range)	
Dimension	Part 1	Part 2
Novelty of Information VAS: 0 = new, 10 = already known	7.8 (0.5–10)	5.5 (0.5–10)
Comprehensibility of Information VAS: 0 = comprehensible, 10 = incomprehensible	0.3 (0–0.7)	0.6 (0–10)
Importance of Information VAS: 0 = important, 10 = not important	4.7 (0.2–8.4)	3.0 (0.8–8.4)
Extent of Information VAS: 0 = too extensive, 10 = not sufficient	5.0 (3.8–7.1)	5.2 (0–8.3)
Impact of Information VAS: 0 = disappointing, 10 = encouraging	5.2 (2.9–7.5)	2.4 (0–9.6)

VAS = Visual Analogue Scale, printed forms, no decimals.

Median novelty of the information was 7.8 for the first part of the program and 5.5 for the second part. However, the wide range of scorings suggests great differences in patients´ knowledge before training. Comprehensibility of the displayed information was rated as very good. Considering the importance of the information delivered, information from the second part was judged more important compared to the information of the first part. Extent of the information presented was judged to be adequate.The first part of the program was neither disappointing nor encouraging for the participants. However, the second part about MS diets and evidence based information on the results of MS-studies was thought to be disappointing (impact of information, mean score 2.4).

At the end of each part of the education program participants were asked about the most relevant information they had received and possible changes that should be made. Information mentioned as valuable was related to understanding the quality of studies (“How to identify good studies”; “ … to critically evaluate study results”), comprehending the influence of fatty acid intake on MS (“No effect of Omega3 /6 fatty acids”), and understanding the lack of knowledge of diet and MS (“Demystification of diets”; “No reliable results on diet and MS”). In addition, participants expressed their disappointment that so little is known about the influence of diet on MS (“that I know nothing”; “all I know is not proven”). Participants made several proposals for future education programs, which can be categorised as follows: Fewer information on studies (“less on studies”; “less theory”), more information on food and nutrition (“more information on food”; “fewer studies, more nutrition, the pros and cons of different foods”), more practical information (“more practical ideas and feasible proposals”), shorter duration (“maybe a little shorter”; “theoretical part a little shorter”). Direct quotes of all comments and proposals are given in S2 Table.

Eight of 13 participants indicated that they would recommend the education program to other PwMS, three were indecisive and 2 would not recommend the program.

## Discussion

Our aim was to design and pilot-test an evidence based patient education program on dietary factors in MS based on the information gathered from a systematic literature review and the data gathered in a web-based survey on dietary habits, perceived effectiveness of dietary interventions and patients`information needs.

About one third of PwMS in our survey believed dietary factors to have an influence on the development of multiple sclerosis and about 60% believed that the course of disease can be influenced by dietary factors. When asked about other factors, the influence of stress/emotions, sports and disease modifying drugs on the course of disease was perceived to be bigger than the influence of diet. Motivation to adhere to special dietary recommendations seemed to follow a realistic assessment of the evidence. 78.3% of PwMS adhering to a diet aimed at improving overall wellbeing and only 8.4% hoped to cure their disease. However, 73% of PwMS adhering to dietary recommendations in our survey aimed at slowing down disease progression. This is in line with findings from an Australian survey (n = 428) that reported that 70% of survey participants aimed at improving overall health and well-being, 60% to tackle fatigue and about 50% to alleviate general MS symptoms when taking supplements or adhering to diets. [13]

Previous studies have shown that diet as well as complementary and alternative medicines (CAM)–which often consist of dietary supplements–is considered highly relevant by PwMS and is widely used for a variety of reasons. [11–13] Although evidence for specific dietary intervention is limited, 42% of PwMS in our survey indicated that they had tried to follow a MS diet or had adhered to special dietary recommendations, which is in line with the findings of an earlier German survey. [12] Attempts to modify fat intake, eat less or no meat and more fish were reported more frequently by PwMS in our survey than specialized MS diets. Accordingly, compared with healthy controls PwMS in our survey were significantly less likely to follow a “Mixed Diet” and significantly more often indicated, that they adhered to a `Mediterranean Diet`.

Intake of dietary supplements was common. In our survey, 76% of both PwMS and control group participants indicated that they had ever used dietary supplements. However, PwMS and controls preferred different types of supplements. Vitamin D, omega-3-fatty-acids and selenium were used more frequently by PwMS whereas controls tended to use Vitamin C and zinc more often. This finding indicates that PwMS tend to take supplements which have been discussed as potentially beneficial for PwMS and are currently under investigation in MS trials, despite limited evidence for a substantial benefit of supplementing Vitamin D or PUFAs. In the Australian survey slightly lower numbers (63.2%) of overall use of supplements have been reported. [13] Data from the German National Nutrition Survey showed that in the general population 27.6% were current users of supplements. [45] Unfortunately, representative data about “ever use” of supplements in the German general population are not available.

The internet was the main source of information on diet and MS used by our survey participants followed by books. Interestingly, although recruitment was performed using the German MS self-help organisation’s website, self-help organisations only played a minor role as information source. These findings differ from those of Leong et al. (2009) who stated that in South Australia conventional health care practitioners and friends and family were the most common sources of information on CAM products and dietary interventions. [13]

Most of the trials included in the systematic review did not report relevant benefits of supplementation on relapse rate or disease progression which is in line with previous reviews by Farinotti et al. (2012) and Jagannath et al. (2010). [6, 10] We found no controlled studies on comprehensive change of dietary patterns as a life style intervention. However, 143 (42%) of the 337 PwMS in our survey, who adhered to a diet or special dietary recommendations, made substantial changes to their eating pattern (e.g. modifying fatty acid intake, eating no meat, adhering to a plant-based, vegetarian diet or a Mediterranean Diet) and 67% of those reported that they felt better or even clearly better when being on a diet. Besides a possible therapeutic effect, the psychological effects of increasing patients’ sense of control by adhering to dietary concepts need to be considered.

The absence of controlled studies for broad dietary approaches leaves patients and caregivers largely clueless and might also have added to the lack of satisfaction with the education program. Our results show that adequately performed studies examining the influence of nutrition on MS are urgently needed in order to support patients in their ability to make informed decisions. Ideally, adequately powered RCTs with comprehensive lifestyle changes as a multimodal intervention [22, 46] and carefully selected outcome measures should be conducted. Taking into account the unpredictability and great variability of disease progression in MS, the conduct of inception cohort studies closely monitoring lifestyle habits (type of diet, level of physical activity) of PwMS over several years could also be considered to improve the amount of available evidence.

In addition to scientific evidence on the influence of diet on MS, patients also requested information on a variety of other topics in a nutritional education program. While our focus was on the methodological problems of dietary studies in MS, future attempts to develop a patient education program on diet and MS should take into account more general aspects of healthy diets.

The survey had a number of limitations. As recruitment was web-based and not population-based, people with a specific interest in the topic might have been more likely to access the web-platform. Additional research should attempt to acquire population-based information. Also representativeness of the control group can be questioned as an unexpectedly high proportion of control group participants indicated to follow a vegetarian diet. Additionally, our questionnaire had some limitations. Most questions were self-developed and not rigorously tested before applying them. This caused misinterpretations of some questions by the participants. For an in depth assessment of dietary habits of PwMS standardized tools for nutritional assessment should be applied in the future. Regarding our evidence based patient education program, a group of 13 consumers is a very limited number to draw inferences. On the other hand this study was meant to be a pilot program to test feasibility and get first, direct feedback from patients. The results could be the starting point for developing a follow-up program which then should be evaluated in different settings and with a substantially higher number of participants.

## Conclusions

The development and pilot-testing of an evidence-based patient education program on nutrition and MS has proven the feasibility of such a program. Patient satisfaction with the program suffered from the lack of evidence and the lack of studies on comprehensive dietary approaches. Further research should focus on generating evidence for the potential influence of lifestyle habits (diet, physical activity) on MS disease course thus meeting the needs of PwMS. Dietary counselling is considered highly relevant by patients. However, with the given evidence patient education needs to mainly focus on the many open questions in the area. Hence, an additional aim should be to enhance patients´ ability to critically appraise the evidence.

## Supporting Information

S1 Checklist PRISMA checklist. (PDF)

S1 Table Experiences with diets and dietary supplements among PwMS–absolute numbers (%). (DOCX)

S2 Table Direct quotes from participants of the evidence-based patient education program. (DOCX)

S1 Text Search terms for the systematic literature search. (DOCX)

S2 Text Pool of categories characterizing EBPI according to Bunge et al., 2010. (DOCX)
