# Supplementary material for: Dietary Interventions in Multiple Sclerosis: Development and Pilot-Testing of an Evidence Based Patient Education Program
Source: PLoS One. 2016 Oct 20;11(10):e0165246. doi: 10.1371/journal.pone.0165246 (PMC5072637; doi:10.1371/journal.pone.0165246)
Supplement: S1 Table — (DOCX) [file pone.0165246.s002.docx]

**S1 Table. Experiences with diets and dietary supplements among PwMS – absolute numbers (%).**

| **Question** |  | **Absolute number (%)** |
| --- | --- | --- |
| Have you ever followed a diet? n=337 | Yes | 143 (42) |
| Have you ever used a supplement? n=337 | Yes | 255 (76) |
| How did you feel on the diet? n=143 | Markedly better | 26 (18) |
|  | Better | 70 (49) |
|  | Unchanged | 43 (30) |
|  | Worse | 4 (3) |
| How did you feel when taking a supplement? n=255 | Markedly better | 16 (6) |
|  | Better | 66 (26) |
|  | Unchanged | 170 (67 |
|  | Worse | 3 (1) |
| Did you stop with the diet? n=143 | Yes | 36 (25) |
| Did you stop taking supplements? n=255 | Yes | 115 (45) |
| After which time did you stop the diet? n=36 | Days | 2 (6) |
|  | Weeks | 5 (14) |
|  | Months | 25 (69) |
|  | Years | 4 (11) |
| After which time did you stop taking supplements? n=115 | Days | 16 (14) |
|  | Weeks | 50 (44) |
|  | Months | 39 (34) |
|  | Years | 10 (9) |
| Why did you stop the diet? (multiple answers possible) n=36 | too much renunciation | 22 |
|  | No effect | 16 |
|  | Too much effort | 10 |
|  | Too expensive | 4 |
|  | Other reasons | 10 |
| Why did you stop taking supplements? (multiple answers possible) n=115 | No effect | 80 |
|  | Too much effort | 20 |
|  | Too expensive | 49 |
|  | Other reasons | 21 |
