# Supplementary material for: Dietary Interventions in Multiple Sclerosis: Development and Pilot-Testing of an Evidence Based Patient Education Program
Source: PLoS One. 2016 Oct 20;11(10):e0165246. doi: 10.1371/journal.pone.0165246 (PMC5072637; doi:10.1371/journal.pone.0165246)
Supplement: S2 Table — (DOCX) [file pone.0165246.s003.docx]

**S2 Table. Direct quotes from participants of the evidence-based patient education program.**

| **Most relevant information mentioned by the participants of the education programme** |
| --- |
| - How to identify good studies - Everything containing omega-acids is good - No tasting of foods in the patient education programme - Intake of polyunsaturated fatty acids - Demystification of diets - That I know nothing. - To critically evaluate study results - Vitamin D, fish oil and food intake - No effect of Omega3 / 6 fatty acids - Study quality - Limited meaningfulness of EDSS values - No association between diet and MS - Studies on fish oil, linoleic acid and vitamin D - Arachidonic acid versus linoleic acid; - Omega 3 /6 have a positive influence - All I know is not proven - No reliable results on diet and MS - Diet may be a factor influencing the MS |
| **Changes to the education programme proposed by participants** |
| - Shorter; maybe a little shorter - Slide on MS in Norway difficult to read - more nutrition and less studies - less on studies - more practical ideas and feasible proposals - tips for fat intake and vitamin D - change of title - fewer studies, more nutrition, the pros and cons of different foods - Information on alternative products - advice for amount of omega3/6 fatty acid intake - theoretical part a little shorter - more information on food - less theory - more specific on nutrition - distribute handout on studies in advance |
