# Supplementary material for: Dietary Interventions in Multiple Sclerosis: Development and Pilot-Testing of an Evidence Based Patient Education Program
Source: PLoS One. 2016 Oct 20;11(10):e0165246. doi: 10.1371/journal.pone.0165246 (PMC5072637; doi:10.1371/journal.pone.0165246)
Supplement: S1 Text — (DOCX) [file pone.0165246.s004.docx]

**S1 Text. Search terms for the systematic literature search.**

(((("fatty acids"[Mesh]) OR ("Minerals"[Mesh]) OR ("Vitamins"[Mesh]) OR ("Nutritional Sciences"[Mesh] OR "Nutrition Assessment"[Mesh] OR "Nutrition Therapy"[Mesh]) OR ("Food"[Mesh] OR "Food and Beverages"[Mesh] OR "Dietary Supplements"[Mesh]) OR ("Eating"[Mesh]) OR ("Diet"[Mesh] OR "Diet Therapy"[Mesh])) AND (multiple sclerosis)) OR (((diet* OR eat* OR nutrition* OR nutrient* OR food*) OR (lipid* OR oil* OR fat* OR fatty acid) OR (omega) OR (PUFA) OR (vitamin OR mineral) OR (diet therapy OR dietary intervention)) AND (multiple sclerosis))) AND ("Epidemiologic Studies"[Mesh] OR "Case-Control Studies"[Mesh] OR "Cohort Studies"[Mesh] OR Case control OR cohort study OR cohort studies OR Cohort analysis OR cohort analyses OR Follow up study OR Follow up studies OR observational study OR observational studies OR Longitudinal OR Retrospective OR Cross sectional OR clinical trial OR randomized controlled trial OR meta-analysis)

The final result was limited to German and English language articles.
