# Supplementary material for: Dietary Interventions in Multiple Sclerosis: Development and Pilot-Testing of an Evidence Based Patient Education Program
Source: PLoS One. 2016 Oct 20;11(10):e0165246. doi: 10.1371/journal.pone.0165246 (PMC5072637; doi:10.1371/journal.pone.0165246)
Supplement: S2 Text — (DOCX) [file pone.0165246.s005.docx]

**S2 Text. Pool of categories characterizing EBPI according to Bunge et al., 2010.**

1. Content of information and meta-information.

2. Quality of evidence.

3. Patient-oriented outcome measures.

4. Presentation of numerical data.

5. Verbal presentation of risks.

6. Diagrams, graphics and charts.

7. Loss- and gain-framing.

8. Pictures and drawings.

9. Patient narratives.

10. Cultural aspects.

11. Layout.

12. Language.

13. Development process.
